# Effects of physical activity on Chinese overseas students’ mental health during the COVID-19: A multi-country cross-sectional analysis

**DOI:** 10.1371/journal.pone.0286321

**Published:** 2023-05-30

**Authors:** Yingjun Nie, Yuanyan Ma, Xiaozhi Yao, Yijia Gao, Xiangping Zheng

**Affiliations:** 1 College of the Physical Education, Wuhan Sports University, Wuhan, China; 2 College of Sports, Huazhong Normal University, Wuhan, China; 3 College of the Arts, Wuhan Sports University, Wuhan, China; Sapienza University of Rome, ITALY

## Abstract

**Background:**

COVID-19 caused severe effects on the psychological well-being of Chinese students overseas (COS). Physical activity (PA) is critical to strengthen immunity, prevent infection, and reduce the psychological burden caused by COVID-19. However, there is a severe lack of effective PA intervention for mental health in most countries, and COS have limited access to mental healthcare during the pandemic.

**Objective:**

We aim to examine the effects of PA on COS’ mental health during the pandemic abroad and to better understand that certain types of PA might be associated with a greater reduction in psychological burdens during the pandemic.

**Methods and results:**

In a multi-country cross-sectional analysis, a questionnaire was distributed to COS living in 37 foreign countries via WeChat Subscription using a snowball sampling strategy. A total of 10,846 participants were included. Descriptive statistics and Binary logistic regression analysis were used for statistical analysis. We found that COS had negative psychology during the pandemic, especially with fear (2.90, 95% CI 2.88−2.92), anxiety (2.84, 95% CI 2.82−2.85), and stress (2.71, 95% CI 2.69−2.73). PA had meaningful effects on reducing COS self-reported mental health burdens (3.42, 95% CI 3.41–3.44) during the pandemic. The largest associations were seen for recreational and home-based PA (i.e., family games, home aerobic exercise), individual outdoor PA (i.e., walking or running, rope skipping), and PA with a duration of 30 to 70 min per session at frequencies of 4 to 6 times and a total of 150 to 330 min of moderate and vigorous intensity per week tends to be an optimal choice during social distancing times.

**Conclusions:**

COS had several poor mental health conditions during the pandemic. The improvement of PA on COS’ psychology was positively effective during the pandemic. Specific types, intensities, durations, and frequencies of PA might have advantages over others for improving COS’ mental health during periods of public health emergencies, and the topic may merit interventional study to reveal multiple factors causing COS’ psychological burdens and enrich the PA forms for all COS’ mental health improvement (i.e., infected, recovered, and asymptomatic COS).

## Background

COVID-19 has undergone several mutations, forcing over 861.7 million students out of school [1], and is less rigorously controlled in the majority of countries worldwide. Colleges in various countries have implemented urgent strategies, such as temporarily closing schools and switching to online classes, to deal with the global public health emergency. And multiple factors, including an increasing number of infected people, asymptomatic patients, social distance, and even delaying graduation, might cause deleterious psychological burdens for students, especially for oversea college students who might experience higher rates of mental health disorders than adults with average age during the pandemic [2]. Now there are about 15.3 million Chinese overseas students (COS) studying abroad [3]. Related research showed that COS experienced varying degrees of psychological challenges during the pandemic (e.g., depression, anxiety, and loneliness) [4, 5], and they were even confronted with allegations of being deemed as potential COVID-19 carriers [5]. For example, recent studies have shown that nearly 50% of COS experienced psychological pressure after the onset of COVID-19 in North America. Moreover, students with serious psychological problems may be more likely to have unhealthy lifestyles, such as drinking alcohol and smoking, and even being reported hopelessness, which could cause a vicious circle [6, 7]. However, COS had limited access to mental healthcare during the pandemic in most countries [3].

There are still many uncertainties in the treatment, infection, and transmission of COVID-19 presently. And these uncertainties are likely to heighten psychological worries. The best way may be to effectively prevent infection and improve self-immunity. Scientific evidence has demonstrated the positive effect of PA on preventing infection and reducing the negative psychological symptoms caused by COVID-19 [8, 9], such as increasing self-mastery to control negative emotions and relaxing mental stress by providing an emotion-communication environment [9]. However, most people experienced restrictions on PA, and their amount of PA decreased sharply due to various worries and many changes of their lifestyles and PA methods [10]. For example, the rate of PA insufficiency among Chinese residents during the outbreak period of Covid-19 increased about 5 times that during the non-epidemic period [11]. And the high prevalence of physical inactivity is a key risk factor for inducing related diseases [12]. Due to being forced to change PA methods and the lack of effective guidance about what forms of PA are beneficial to health status, most people are confused about whether PA is significant and how to participate in PA to their well-being, and even severely lack of PA guidance and mitigation strategies to advance the knowledge and role of PA for mental health in most countries during the epidemic [13], and there also is a severe lack of effective PA intervention for mental health [11]. Therefore, finding effective PA to achieve significant benefits for COS’ psychological well-being, as well as a better understanding of the relationship between certain PA and mental health improvement, is critical during the pandemic.

Given that the degree of psychological improvement may vary as a function of different PA, for instance, recreational group PA may be of higher effectiveness than individual PA in emotional relaxation and stress relief [14], and moderate intensity PA may lead to better mental health improvements including relief of depression and anxiety during the pandemic [11], we intended to explore the answers to the following questions: what types of PA are beneficial to psychological improvement? Whether certain forms of PA may be better than others during the epidemic? As a result, we used a multi-country representative sample survey to discover the relationship between PA and COS’ psychological well-being during the pandemic. Investigations such as the present study are essential for providing important references and evidence to promote PA and timely crisis-oriented psychological PA interventions for COS during the pandemic.

## Methods

### Study population

COS are mainly distributed in North America, Western Europe, Africa, Oceania (Chinese Ministry of Education, 2020). Therefore the study used a snowball sampling strategy to recruit COS not infected with COVID-19, who lived in 37 foreign countries across Asia, North America, Oceania, Europe, and Africa during the pandemic, and a questionnaire was distributed via the WeChat with high usage rate of all young people in China. To avoid the exclusion of students who do not use WeChat during the COVID-19, some organizers of Chinese student societies in various college of oversea countries were invited to participate in questionnaire distribution using their social platforms, including enrollment via both online and offline. Through these ways, ensure that COS from different countries participated and to maximize the diversity and representativeness of the COS participating in the survey.

Individuals were asked to recall their mental health and PA during the pandemic. The data collection period was from December 1, 2020, to February 28, 2021. Furthermore, full ethical approval was obtained from Wuhan Sports University. All participants gave their informed consent in writing while completing the questionnaire.

### Survey

From April to September 2020, the COVID-19 pandemic reached its peak globally, and over 10 million confirmed cases and 430 thousand deaths were reported in the above 210 countries and regions (WHO, 2020). Therefore, this study focuses on COS’ PA and mental health in this period, and all the participants were asked to recall their PA and mental health status from September 1^st^ to 30^th^, 2020.

PA data were collected by an original self-report scale of IPAQ-C (the Chinese version of the International PA Questionnaire). The Cronbach’s alpha coefficient was 0.89, and the test-retest reliability was 0.82, indicating the good reliability of the questionnaire. All data were managed and screened according to standard methods and the guidelines for data processing and analysis in the IPAQ. COS were asked to recall the type, intensity, frequency, and duration of various PA that they engaged in during the period of September 1^st^ to 30^th^, 2020.

Mental health was assessed using the 50-item Self-evaluation Table for COS’ mental health during the pandemic (a semi-standardized test), which was compiled based on Symptom Checklist 90 (SCL-90). The Cronbach’s alpha coefficient was 0.92, and the test-retest reliability was 0.80, indicating good reliability of the questionnaire. COS were asked to recall the improvement of PA in their psychology during the period of September 1^st^ to 30^th^, 2020, including anxiety, fear, depression, somatization, and stress. The Cronbach’s alpha coefficients for each sub-scale were 0.87 for anxiety, 0.91 for fear, 0.92 for depression, 0.88 for somatization, and 0.90 for stress, respectively. The test-retest reliabilities were 0.77 for anxiety, 0.79 for fear, 0.82 for depression, 0.81 for somatization, and 0.83 for stress, respectively. Both scores indicate good reliability of all sub-scales. Each item was rated on a five-point Likert scale, ranging from 1 (very slightly or not at all) to 5 (extremely), to indicate the extent to which the participants felt that each psychology item pertained to them.

### Statistical analysis

SPSS software 26.0 (IBM Inst., Chicago, IL, USA) is employed to analyze all data. Descriptive statistics (i.e., percentages, 95% CI, means, and standard deviations) were calculated for categorical variables and continuous variables to reflect the demographic characteristics of the survey population. Binary logistic regression analysis was conducted to reveal the effect of PA on improving COS’ psychology during the pandemic.

## Results

### Survey respondents

The final analysis included 10,846 participants from 138 institutions in 37 countries (Table 1), ranging in age from 18 to 49 years old (Mean = 26.3 years old, Standard Deviation = 8.46 years). The participants included 5225 undergraduates, 2174 postgraduates, 1885 Ph.D. students, and 1562 visiting scholars, including 5200 females and 5646 males (Table 2).

**Table 1:** Distribution of respondents in counties.

Countries	Count (percent)	Countries	Count (percent)	Countries	Count (percent)
**Canada**	616 (5.67%)	Estonia	52 (0.47%)	United Kingdom	1022 (9.42%)
**America**	1279 (11.79%)	Netherlands	170 (1.56%)	India	32 (0.3%)
**New Zealand**	872 (8.03%)	Finland	256 (2.36%)	Uzbekistan	19 (0.17%)
**Australia**	1321 (12.17%)	Bosnia and Herzegovina	132 (1.22%)	Turkey	21 (0.19%)
**Cote D’Ivoire**	52 (0.47%)	Russia	879 (8.1%)	Thailand	20 (0.18%)
**Lesotho**	36 (0.33%)	Italy	321 (2.96%)	Malaysia	16 (0.15%)
**Tanzania**	26 (0.23%)	Czech Republic	89 (0.82%)	Kazakhstan	49 (0.45%)
**Sudan**	39 (0.35%)	Albania	65 (0.6%)	Korea	542 (5%)
**Mozambique**	26 (0.23%)	Germany	562 (5.18%)	Georgia	32 (0.29%)
**Togo**	93 (0.85%)	Hungary	102 (0.94%)	Philippines	12 (0.11%)
**Greece**	21 (0.19%)	France	467 (4.31%)	Japan	598 (5.51%)
**Cyprus**	16 (0.14%)	Poland	369 (3.4%)	Singapore	554 (5.11%)
**Montenegro**	10 (0.09%)				

**Table 2:** Demographic characteristics of respondents.

states	Frequency	Education level	Frequency	Age (years)	Frequency
**Asia**	1895(17.47%)	Undergraduate	5225(48.17%)	18–24	3994(36.82%)
**North America**	2193(20.22%)	Postgraduate	2174(20.04%)	25–29	3086(28.45%)
**Oceania**	1953(18.01%)	PhD student	1885(17.38%)	30–39	2777(25.60%)
**Europe**	4533(41.79%)	Visiting scholar	1562(14.40%)	40–49	989(9.12%)
**Africa**	272(2.51%)				

### The influence of COVID-19 on COS’ mental health

The COVID-19 pandemic has significantly negative effects on COS’ psychology. During the pandemic, COS’ psychology was affected to varying degrees (Table 3) with fear (2.90, 95% CI 2.88−2.92), anxiety (2.84, 95% CI 2.82−2.85), stress (2.71, 95% CI 2.69−2.73), depression (2.41, 95% CI 2.39−2.43), and somatization (2.21, 95% CI 2.19−2.22). Accordingly, fear, anxiety, and stress might be more seriously affected.

**Table 3:** COS’ psychology status during the pandemic.

	M±SD	Variance	95% CI (LL)	95% CI (UL)	CV
**Fear**	2.90 ± 0.93	0.87	2.88	2.92	32.16%
**Anxiety**	2.84 ± 0.93	0.87	2.82	2.85	32.93%
**Stress**	2.71 ± 1.02	1.04	2.69	2.73	37.77%
**Depression**	2.41 ± 0.96	0.92	2.39	2.43	39.72%
**Somatization**	2.21 ± 0.86	0.74	2.19	2.22	38.99%

M = mean; SD = standard deviation; CI = Confidence Interval; CV = coefficient of variation. The negative effects of COVID-19 on COS’ psychology were estimated with lower limits (LL) and upper limits (UL) of 95% confidence interval (CI).

### The influence of PA on COS’ mental health

#### The positive effect of PA on COS’ mental health

This study found that PA had positive effects on reducing COS’ mental health burdens (3.42, 95% CI 3.41−3.44) during the pandemic (Table 4), including somatization (3.31, 95% CI 3.29−3.33), anxiety (3.53, 95% CI 3.51−3.54), depression (3.22, 95% CI 3.20−3.24), stress (3.54, 95% CI 3.52−3.56) and fear (3.52, 95% CI 3.51−3.54). And their correlations with PA were respectively 0.51, 0.53, 0.65, 0.58 and 0.62, all greater than 0.5.

**Table 4:** The positive influence of PA on COS’ psychology status.

	M±SD	Variance	95% CI (LL)	95% CI (UL)	CV
**Improved somatization**	3.31±0.99	0.99	3.29	3.33	30.18%
**Decreased anxiety**	3.53±0.90	0.81	3.51	3.54	25.56%
**Decreased depression**	3.22±0.94	0.89	3.20	3.24	29.33%
**Decreased stress**	3.54±0.92	0.85	3.52	3.56	25.98%
**Decreased fear**	3.52±0.87	0.76	3.51	3.54	24.73%
**Improved mental health**	3.42±0.81	0.66	3.41	3.44	23.75%

M = mean; SD = standard deviation; CI = Confidence Interval; CV = coefficient of variation. The positive effects of PA on COS’ mental health were estimated with lower limits (LL) and upper limits (UL) of 95% confidence interval (CI). The type, intensity, frequency, and duration of various PA was self-reported and was included in the logistic regression as a continuous variable.

#### Influence of different PA types on improving mental health

**Fig 1:**
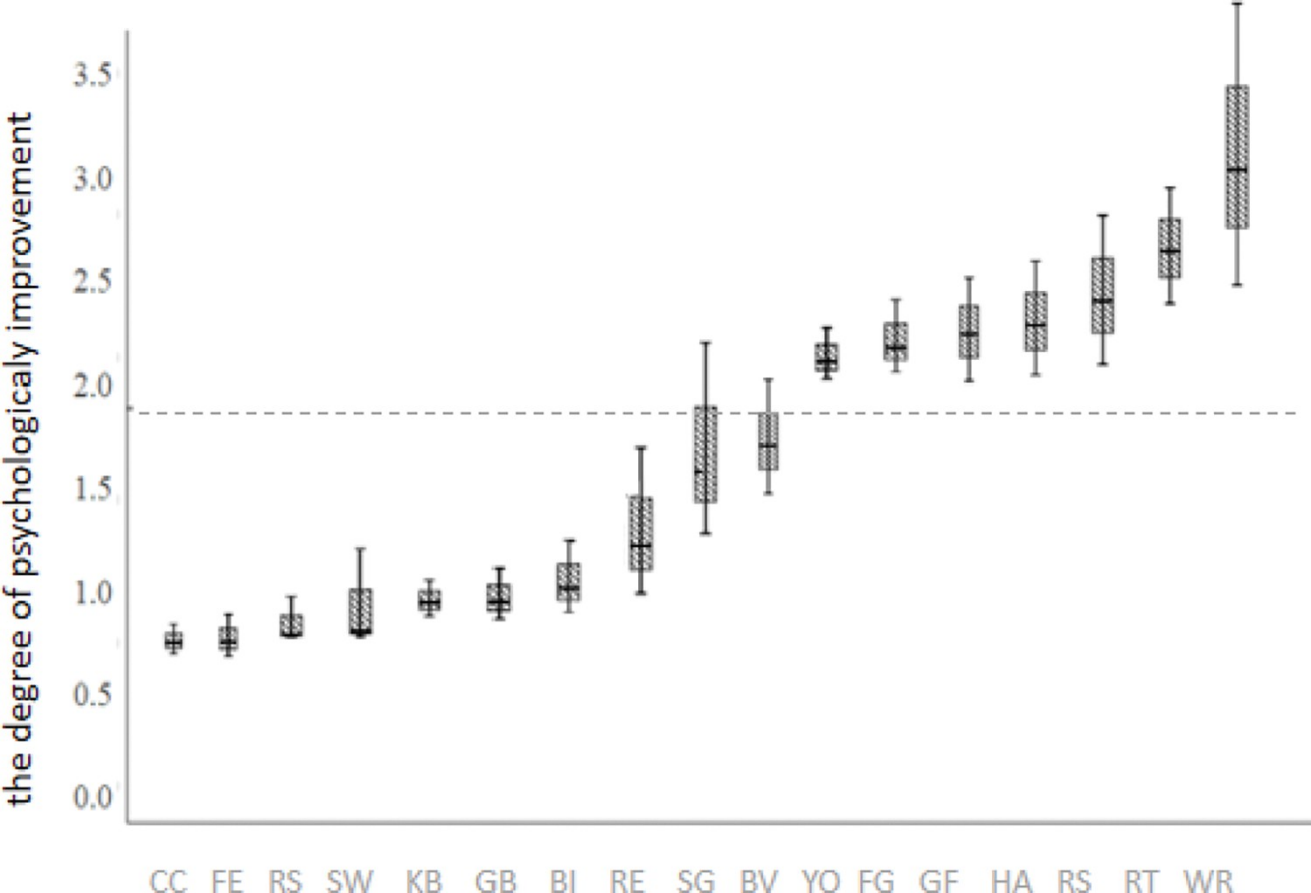
The effect of different PA types on improving COS’ mental health.

Binary logistic regression was used to analyze the effect of PA types on improving mental health during COVID-19. The results showed that some types of PA had a greater improvement in mental health burden than others during the pandemic (Fig 1). Including Yoga (2.10, 95% CI 1.85−2.35), Family games (2.20, 95% CI 2.06−2.41), Gymnastics, Freehand exercises, Dancing (2.25, 95% CI 1.90−2.51), Home aerobic exercise (2.32, 95% CI 2.11−2.47), Rope skipping (2.43, 95% CI 2.21−2.77), Resistance training (2.62, 95% CI 2.16−3.18), and Walking or running (3.13, 95% CI 2.61− 3.67) (Table 5). Walking or running (3.13, 95% CI 2.81−3.45) have the greatest effect on mental health, Rope skipping exert the greatest influence on somatization (3.12, 95% CI 2.90−3.34), Home aerobic exercise on anxiety (2.81, 95% CI 2.52−3.10) and fear (3.05, 95% CI 2.87−3.23), Gymnastics, Freehand exercises, Dancing on depression (3.23, 95% CI 3.01−3.45), Resistance training on stress (2.93, 95% CI 2.63−3.23) (Table 6).

**Table 5:** The effect of PA types on improving mental health.

PA types	SE	Z	P	OR	95% CI	
					Lower	Upper
**WR = Walking or running**	1.41	-3.44	0.001	3.13	2.61	3.67
**RT = Resistance training**	0.10	-8.55	0.000	2.62	2.16	3.18
**RS = Rope skipping**	0.15	-6.77	0.000	2.43	2.21	2.77
**HA = Home aerobic exercise**	0.14	-10.46	0.000	2.32	2.11	2.47
**GF = Gymnastics, Freehand exercises, Dancing**	0.11	-14.12	0.000	2.25	1.90	2.51
**FG = Family games**	0.13	-8.75	0.000	2.20	2.06	2.41
**YO = Yoga**	0.12	-12.78	0.000	2.10	1.85	2.35
**BV = Basketball, volleyball, or football**	0.17	-1.61	0.106	1.79	1.54	1.96
**SG = Sensory-motor games and whole body vibration**	0.36	-5.53	0.000	1.63	1.53	1.78
**RE = Random exercise**	0.19	-9.22	0.000	1.45	1.28	1.65
**BI = Bicycle**	0.25	-12.04	0.000	1.31	1.21	1.51
**GB = Golf ball**	1.45	-0.89	0.37	1.25	1.10	1.40
**FB = Fight drill, boxing**	0.34	-7.74	0.000	1.20	0.105	0.13
**SW = Swim**	0.34	-8.74	0.000	1.05	0.90	1.32
**RS = Roller-skating**	1.41	-3.99	0.000	0.93	0.70	1.26
**FE = Fencing**	1.55	0.02	0.983	0.81	0.69	1.03
**CC = Chess classes**	0.35	-4.91	0.000	0.79	0.50	0.97

SE = Standard Error; Z = Z-Score; P = P-value; OR = Odds Ratio; CI = Confidence Interval. The effect of PA types on improving mental health was estimated with lower limits (LL) and upper limits (UL) of 95% confidence interval (CI). OR was introduced to reflect the relative merits of different types of PA.

**Table 6:** The types with the greatest effect on different mental health indicators.

Mental health improvement	PA types with the greatest effect	SE	Z	P	OR	95% CI	
						Lower	Upper
**Improved mental health**	WR = Walking or running	1.41	-3.44	0.001	3.13	2.81	3.45
**Improved somatization**	RS = Rope skipping	1.12	-7.56	0.000	3.12	2.90	3.34
**Decreased anxiety**	HA = Home aerobic exercise	0.45	-6.54	0.000	2.81	2.52	3.10
**Decreased fear**	HA = Home aerobic exercise	0.63	-9.73	0.001	3.05	2.87	3.23
**Decreased depression**	GF = Gymnastics, Freehand exercises, Dancing	0.19	-8.36	0.000	3.23	3.01	3.45
**Decreased stress**	RT = Resistance training	0.21	-7.23	0.000	2.93	2.63	3.23

SE = Standard Error; Z = Z-Score; P = P-value; OR = Odds Ratio; CI = Confidence Interval. The effect of PA types on improving mental health was estimated with lower limits (LL) and upper limits (UL) of 95% confidence interval (CI).

#### Influence of different PA intensities on improving COS’ mental health

**Fig 2:**
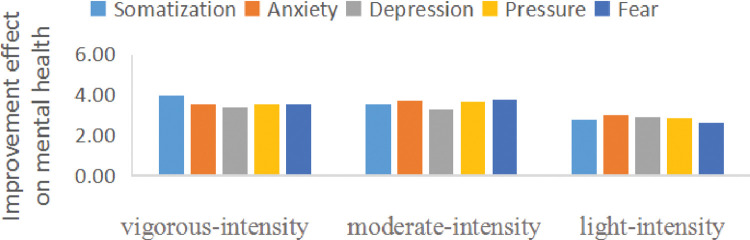
The effect of different PA intensities on improving COS’ psychology.

According to the standards of the IHO [15], PA intensity is divided into light intensity (approximately 35−59% of the maximum heart rate), moderate intensity (approximately 60−69% of the maximum heart rate), and vigorous intensity (greater than or equal to 80% of the maximum heart rate). We found that all types of PA intensity had positive effects on improving COS’ mental health, and the effect of moderate-intensity exercise (3.57, 95% CI 3.24−3.75, p<0.05) and vigorous-intensity (3.58, 95% CI 3.29−4.06, p<0.05) on improving COS’ psychology might be more effectual than that of light-intensity (3.12, 95% CI 2.76−3.30, p<0.05) during the COVID-19 (Fig 2).

#### Influence of PA duration per session on improving COS’ mental health

**Fig 3:**
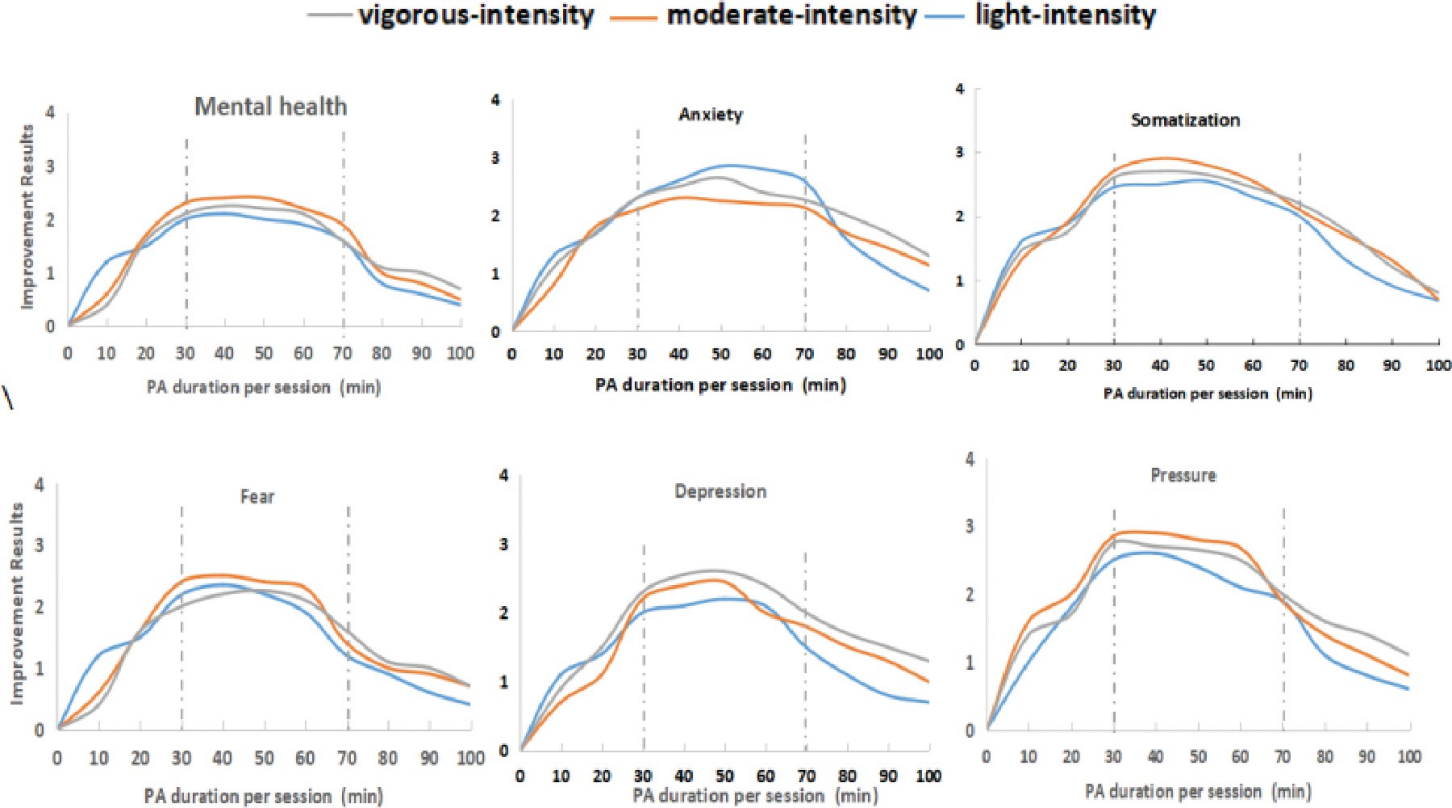
The effect of different PA duration per session on improving COS’ psychology.

Overall, the improvement rate of different psychological conditions might reach a climax when a PA duration per session approaches from 30 to 70 min (Fig 3). However, for COS who engaged in less than 10 min or more than 90 min of PA per session, the improvement of psychological conditions might be significantly lower during the pandemic.

**Fig 4:**
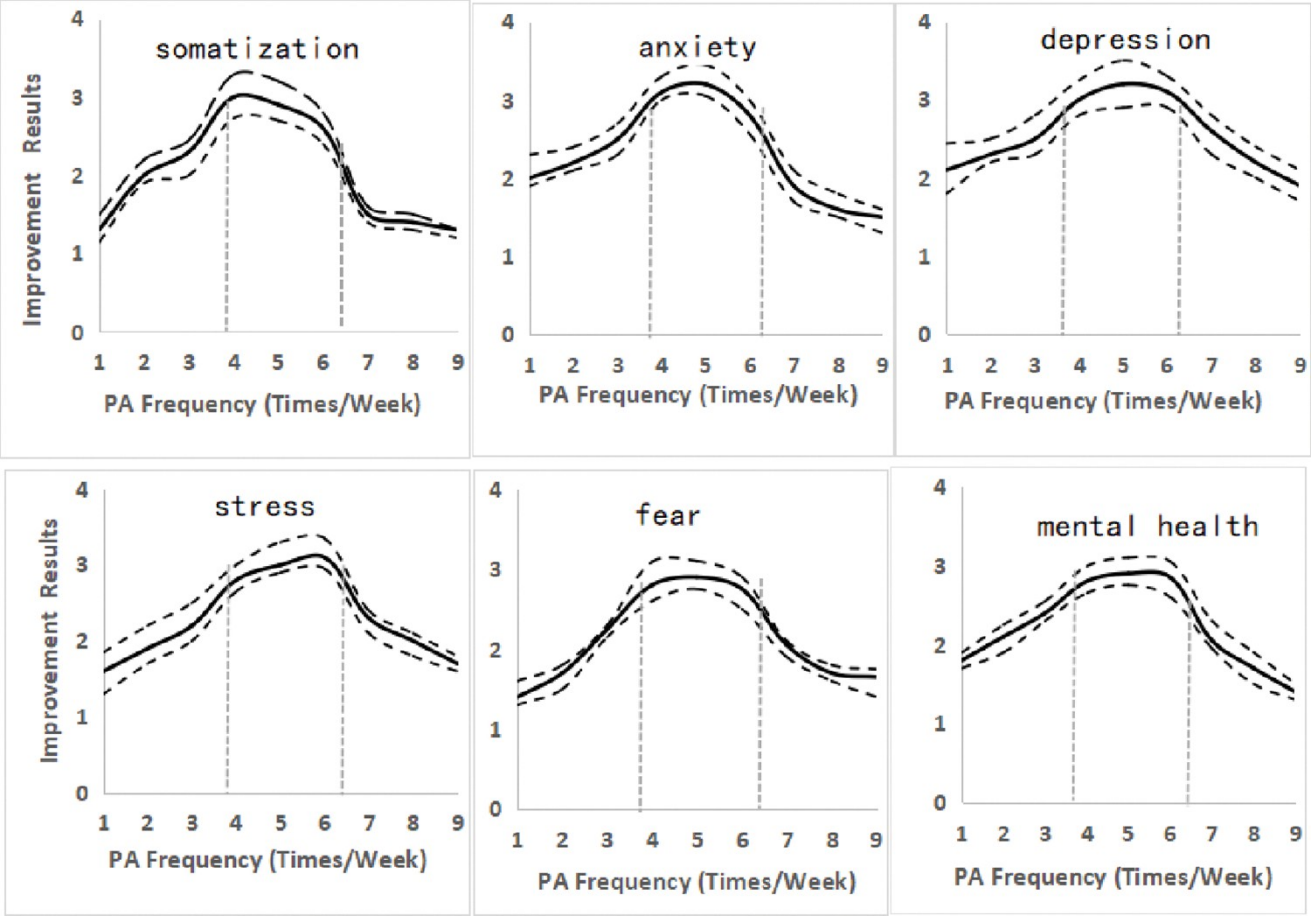
The different effects of PA frequency per week on improving COS’ psychology.

According to Fig 4, a PA frequency of 4 to 6 times per week (p<0.05) might be better for ameliorating mental conditions. On the contrary, less than 2 times or more than 8 times of PA per week might be associated with a lower effect on psychological improvement.

**Fig 5:**
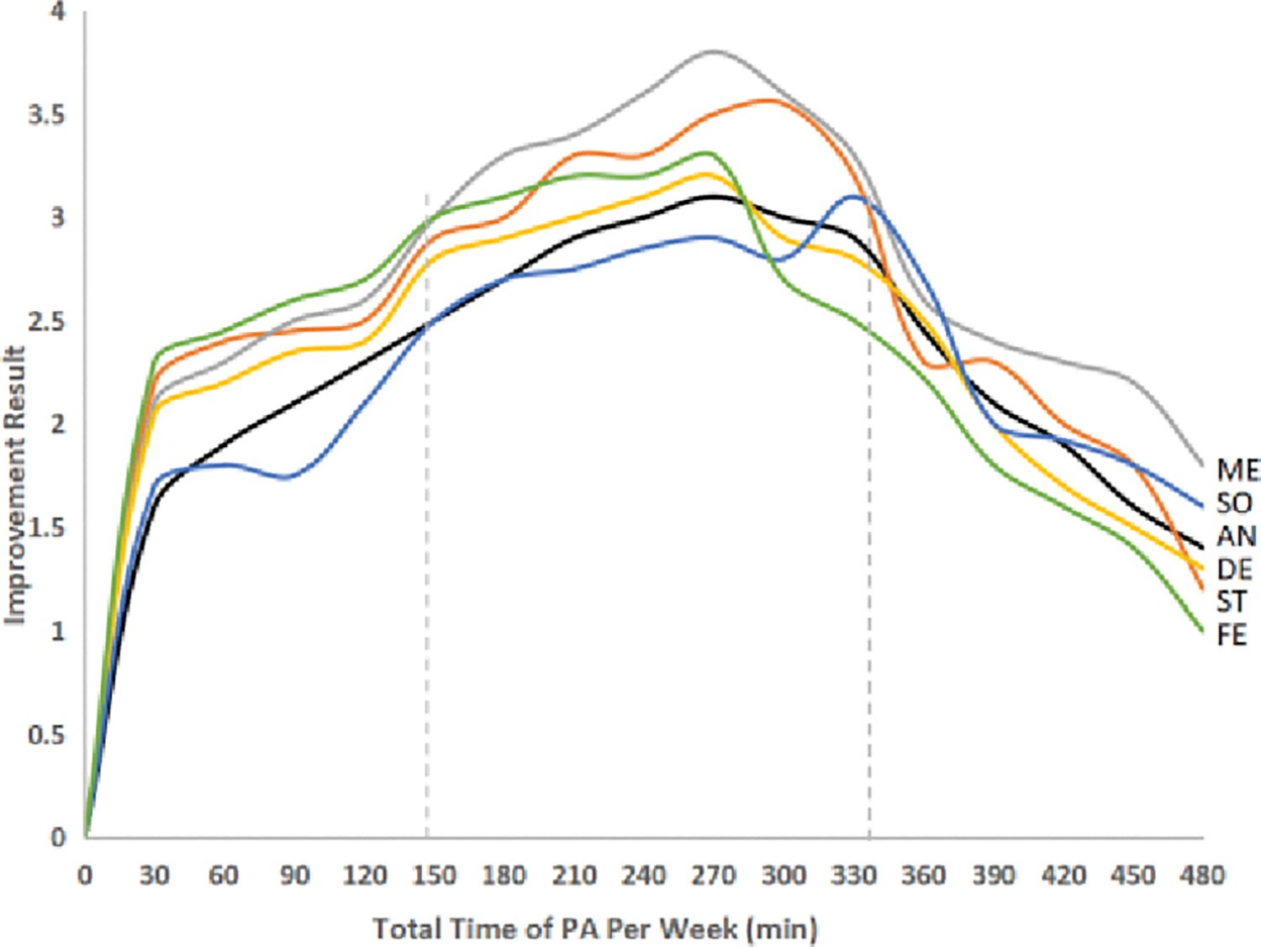
The different effects of weekly total PA on improving COS’ psychology. (ME = mental health; SO = somatization; AN = anxiety; DE = depression; ST = stress; FE = fear).

For all types of mental health burdens, better-improving psychology was seen for COS who engaged in a total time of PA per week from 150 to 330 min during the pandemic (Fig 5, p<0.05), regardless of intensity. And lower improving psychology was seen for COS who engaged in less than 30 min or more than 360 min of PA per week during the pandemic.

## Discussion

### Analysis of the impact of COVID-19 on mental health

This study showed that COVID-19 had harmed COS’ psychological status. During the pandemic, COS living abroad experienced different forms of psychological challenges, including somatization, anxiety, depression, stress, and fear. This phenomenon may be shared with all overseas students during the pandemic. Overall, prolonged school closure, cancellation of classes, new modes of instruction, daily lifestyle changes, and much more might accentuate or creates new stressors for COS’ mental health who lived aboard during the pandemic [16].

Firstly, epidemic severity might be the most important factor causing negative psychological impacts. COVID-19 has undergone several mutations. Various factors could lead to more serious psychological symptoms of COS living abroad, including the most possible of being asymptomatic carriers for youth [17], the growing numbers of confirmed COVID-19 cases and deaths, the increasing population being infected after vaccination, and some Covid-19 recovered persons being re-infected. The worry of being infected positively correlates with depression, anxiety, and virus aversion among COS. Besides, COS might face discrimination, alienation, and isolation in some countries due to being deemed as potential SARS-CoV-2 carriers [18]. Research reported that since the WHO declared the COVID-19 outbreak in March 2020, stigma and discrimination against the Chinese has been on the rise [19], which may lead to stress, anxiety, and fear of COS.

Secondly, the worry about academic performance might positively correlate with COS’ psychological problems during COVID-19, and mental health issues also are the leading impediment to academic success, which could cause a vicious circle [4]. For example, prolonged school closures and class postpones might have negative effects on the contact with lecturers and the support from classmates, then lead to increased fear of passing their examinations [20]. In addition, disruptions of their research projects and internships during school closure might delay their graduation and undermine their competitiveness in the job market, which could cause negative effects on COS’ mental health during COVID-19. Furthermore, many colleges switched to online classes during the COVID-19 pandemic, but some COS were unfamiliar with the new online learning methodology, and the virtual classes were stressful for them to adapt to web-based teaching platforms and technology rapidly [21]. These procedures for passing course exams and presenting their work via online platforms might also be stressful for them [22]. Moreover, the significant increase in screen time mainly being spent on the computer and Internet usage may be an additional source of COS’ psychological symptoms [23].

Thirdly, the epidemic prevention environment of the host government, including infection control policies and preventive measures, are generally different among counties, which may be additional challenges to assessing the impact of COVID-19 on COS’ psychological well-being. In fact, COVID-19 has caused a massive burden on governments and individuals around the world. Governments in various countries have implemented urgent national containment policies to prevent the spread of the pandemic. However, the spread of the pandemic is not effectively controlled, and the number of COVID-19 cases and deaths is increasing significantly in some countries [11]. The uncontrollability of COVID-19 likely arouses COS’ worry about their health [24], and COS tend to experience more anxiety if someone they know is tested positive for COVID-19 [2]. In addition, populations differ on many dimensions between and within countries, such as socioeconomic status, views about COVID-19, and religious beliefs, which all may lead to inconsistent and even conflicting views on COVID-19 and various epidemic-prevention measures between COS and the locals, such as whether to wear a mask outdoor and whether to get vaccinated. That is to say, if COS cannot accept or adapt to the epidemic prevention environment of the host government, their level of anxiety likely increases.

Finally, obstruction of return trips might make some COS more prone to psychopathology. Because some counties closed or reduced international air at the beginning of the COVID-19 outbreak or at spikes occur in COVID-19 cases, it was difficult for many COS to buy air tickets to China on time. Some COS even have concerns about infection, COVID-19 tests, and fears of being quarantined on the way back to China. Moreover, some COS might also struggle with the expensive cost of returning to China [25].

### Analysis of the PA on improving COS’ mental health

This study showed that adequate PA had a positive and significant effect on improving COS’ mental health during the pandemic, and the result of mental health improvement may vary with different PA types, intensities, durations, and frequencies. Related research also reported that PA might reduce the mental health burden caused by COVID-19 [9]. In the context of the worry of being infected and the social distancing strategy, most COS might likely choose the recreational and home-based PA, and individual outdoor PA, which might have a greater benefit to emotional relaxation and psychological burden relief. Our other research showed that most people participated in Chinese traditional sports in home quarantine and social isolation during the COVID-19 outbreak in China, such as Chinese martial arts, Taijiquan and Qigong [8], and scientific evidence shows that Chinese martial arts are of great benefit for emotion control, and mental improvement, as well as for the prevention, treatment, and rehabilitation of COVID-19, due to a sequence of movements and postures with the regulation of the breathing rhythm and pattern, musculoskeletal stretching and relaxation [26–28]. Nevertheless, few COS seem to participate in Chinese martial arts during the pandemic. It may be necessary to strengthen the education and dissemination of Chinese martial arts for COS, and the establishment of a library of traditional Chinese sports may be an effective strategy to improve COS’ mental health.

The form of PA is the key to creating pleasant psychology. This study showed the form of COS’ PA was relatively simple and mainly focused on individual PA during the pandemic. Countries differ politically and economically, the strategies adopted and the difficulties faced vary within each country, as well as the pandemic severity. For example, the UK adopted a policy of herd immunity; students were required to move out of on-campus housing in some colleges in the USA. Consequently, most COS experienced different restrictions on PA and were forced to change their PA methods during the pandemic [29]. Thus, positive guidance and strategies likely need to be tailored to promote COS’ PA and timely crisis-oriented psychological PA interventions for COS during the pandemic. For example, in the absence of contact with lecturers and support from classmates, the use of network visualization to participate in collective PA with friends or family who are in China may provide COS with emotional communication. In addition, it may be potentially useful for COS to take other professional PA like respiratory exercise, which has been confirmed to have great effects on the prevention, treatment, and rehabilitation of COVID-19 [30].

Related research reported the mental health burden might vary as a function of PA type and there may be an inverted U-shaped relationship between mental health improvement and PA duration as well as PA frequency [31], and unreasonable and inappropriate PA may even damage the immune system and lead to poor mental health [11]. Our study showed the strongest associations between COS’ mental health and PA with a duration of 30 to 70 min per session, frequencies of 4 to 6 times, and a total of 150 to 360 min of moderate and vigorous intensity per week during the pandemic. In our previous study, we found that most people between 12 to 69 years old primarily participated in moderate-intensity PA with 30–60 min duration, 3 to 5 times and a total of 120 to 270 min of PA per week, which was associated with better mental health in China during the COVID-19 outbreak when home quarantine increased severe restrictions and inconvenience on PA [11]. Many COS between 18 to 49 years old tended to participate in moderate and vigorous intensity outdoor PA abroad during the pandemic. The inconsistent findings might be due to differences in the PA environment and participants’ ages. Research also showed that people of different ages may have different PA intensity levels and content during the COVID-19 outbreak period [14].

The IHO also recommended that the population engage in a total of 150 min of moderate-intensity aerobic PA or 75 min of high-intensity PA per week [32]. However, prolonged strenuous PA has the potential risk of suppressing immune system function, leading to increased susceptibility of infections and the appearance of latent viral reactivation [33, 34]. Our investigation showed that more than 8 times or 360 min of PA per week might be associated with a lower effect on psychological improvement for COS during the pandemic. Research also reported PA with more than 6 hours per week or more than 2 hours per session may cause worse mental health burdens [15]. Thus the degree of effectiveness of PA on COS’ mental health may be related to specific PA intensities, durations, and frequencies during the pandemic. Therefore, during the pandemic, it may be meaningful and necessary for COS to significantly strengthen comprehensive PA monitoring, including duration and frequency, especially the high-intensity PA.

### Limitations and prospect of research

This study had several limitations. Firstly, although the questionnaires were distributed through the populous COS social media platform to maximize the diversity and representation of the subjects, the demographic distribution of participants was certain unevenness and incompleteness. Secondly, the study sample was targeted as individuals who are not infected with COVID-19. For infected people, recovered people, and asymptomatic patients with COVID-19, further research is necessary and meaningful concerning effective PA and mental health. Thirdly, the study tended to rely largely on online self-reported questionnaires to obtain the intensity and content of PA and psychological status, reporting bias cannot be excluded. Finally, this study, as a cross-sectional data study across countries, may not capture differences in PA and mental health outcomes across countries with different epidemic policies over the same time period at the time the results were obtained.

## Conclusions

COS living aboard showed different degrees of psychological challenges during the pandemic, especially fear, anxiety, and stress. Appropriate PA was associated with meaningful improvements in self-reported mental health issues, such as stress, anxiety, depression, and somatization. It is suggested that multiple factors may cause COS’ psychological burdens, such as the epidemic severity, infection rate and deaths, the effectiveness of epidemic prevention policies, and medical readiness in host countries. Clearly, specific types, intensities, durations, and frequencies of PA were found to be more effective for improving mental health during the pandemic. The largest associations were seen for recreational and home-based PA, and individual outdoor PA, with a duration of 30 to 70 min per session, as well as PA at frequencies of 4 to 6 times and nearly 150 to 330 min of moderate and vigorous intensity per week. Above all, it is reassuring for COS to experiment with more PA forms and strength monitoring of PA volume in such intense circumstances.
